# Real-world impact of treatment on growth in children with X-linked hypophosphatemia

**DOI:** 10.1210/clinem/dgag136

**Published:** 2026-03-31

**Authors:** Seiji Fukumoto, Dieter Haffner, Erik A Imel, Keiichi Ozono, Maria Luisa Brandi, Haruka Ishii, Zhiyi Li, Kerry Sandilands, Paul Joos-Vandewalle, Cheuk Lee, Masanori Kanematsu, Keith P McCullough, Thomas O Carpenter

**Affiliations:** Department of Medicine, Tamaki-Aozora Hospital, Tokushima 779-3125, Japan; Department for Pediatric Kidney, Liver, Metabolic and Neurological Diseases, Hannover Medical School, 30625 Hannover, Germany; Departments of Medicine and Pediatrics, Indiana University School of Medicine, Indianapolis, IN 46202, USA; Department of Pediatrics, Center for Promoting Treatment of Intractable Diseases, ISEIKAI International General Hospital, Osaka 530-0052, Japan; Department of Endocrinology, University Vita-Salute San Raffaele, 20132 Milan, Italy; FIRMO Foundation, 50129 Florence, Italy; Medical Affairs Department, Kyowa Kirin Co. Ltd., Tokyo 100-0004, Japan; HEOR, Rare Disease (Medical Affairs), Kyowa Kirin North America, Princeton, NJ 08540, USA; Outcomes Research, Kyowa Kirin International Plc, Marlow SL7 1HZ, UK; Global Medical Affairs, Kyowa Kirin Co. Ltd., 28036 Madrid, Spain; Global Medical Affairs, Kyowa Kirin International Plc, Marlow SL7 1HZ, UK; Medical Affairs Department, Kyowa Kirin Co. Ltd., Tokyo 100-0004, Japan; Arbor Research Collaborative for Health, Ann Arbor, MI 48104, USA; Department of Pediatrics, Section of Endocrinology, Yale School of Medicine, New Haven, CT 06510, USA

**Keywords:** X-linked hypophosphatemia (XLH), phosphate-regulating endopeptidase homolog, X-linked (PHEX) gene, fibroblast growth factor 23 (FGF23), height, growth velocity, real-world evidence

## Abstract

**Context:**

X-linked hypophosphatemia (XLH) is a rare, genetic, progressive, lifelong disorder manifest by impaired growth and disproportionate short stature. Burosumab, a monoclonal antibody against fibroblast growth factor 23, is approved for treating patients with XLH.

**Objective:**

To understand the impact of burosumab treatment on growth in a real-world setting.

**Design:**

Interim data from 3 ongoing, real-world observational studies (NCT03651505, NCT03193476, and NCT03745521), were unified into a single study (APEX).

**Setting:**

Outpatient clinics.

**Patients:**

Children aged 2-17 years with XLH.

**Intervention(s):**

Subcutaneous burosumab vs oral phosphate salts and active vitamin D or no treatment.

**Main Outcome Measure(s):**

Retrospective and prospective height data from children enrolled in APEX were analyzed to provide long-term estimation of the impact of treatment on growth. Growth and height were estimated by a mixed regression model with separate models for children and adolescents.

**Results:**

In total, 641 participants were analyzed (402 female) with 498 burosumab-treated and 143 burosumab naïve. Median (interquartile range [IQR]) enrollment ages were 8 (5-12) years for burosumab-treated and 10 (4-14) years for burosumab-naïve participants. Burosumab-treated participants experienced improved growth over burosumab-naïve participants (additional growth velocity: 0.085 Z-score/year for children [*P* < .0001]; 0.121 Z-score/year for adolescents [*P* < .0001]). Modeling predicted greater adult height among burosumab-treated participants.

**Conclusion:**

Burosumab had a robust, positive association with improved growth outcomes in male and female children and adolescents with XLH. Modeling based on a median of 3.3 years of follow-up predicted burosumab can support improvement of growth that will likely result in greater adult height.

X-linked hypophosphatemia (XLH) is a rare, progressive, and lifelong metabolic bone disorder and the most common form of heritable rickets (1-4). XLH is caused by pathogenic variants in the phosphate-regulating endopeptidase homolog, X-linked (*PHEX*) gene, which results in elevated levels of fibroblast growth factor 23 (FGF23), chronic hypophosphatemia, and impaired bone and tooth mineralization (1, 3, 5). In children, this typically leads to delayed motor development, rickets, and lower limb deformities (1, 3, 6-9). Body length is within normal ranges at birth, but effects on bone development usually become evident in infancy, resulting in reduced growth with disproportionate short stature in children and adults (1, 3, 7, 10-12). Treatment with frequent doses of phosphate salts and active vitamin D analogs (Pi/D) may improve growth in patients with XLH, particularly when initiated early (eg, <1-2 years of age) (10-13), but does not normalize adult height (1, 3, 10-15).

Burosumab is a recombinant fully human monoclonal antibody against FGF23 (16, 17). Burosumab is approved for treating XLH in children and adults (18-20). Randomized controlled trials (RCTs) of burosumab demonstrated improved phosphate homeostasis, effective treatment of rickets in children, and healing of pseudofractures in adults, with an acceptable safety profile (16, 21-23). Burosumab modestly improved height Z-scores in children compared with baseline and those treated with Pi/D (21, 23, 24). Evidence from real-world studies indicates that switching from Pi/D to burosumab treatment improved height Z-scores from the time of burosumab initiation over a follow-up ranging from 1 year up to a median of 3.3 years (25-30). However, this effect on growth was not apparent in all studies (31, 32). Several clinical and real-world studies support earlier initiation of burosumab to improve height outcomes (24, 27, 30), although these studies are limited by small sample sizes, and long-term outcomes are currently unavailable. Additional studies are required to augment the current body of evidence.

The Advancing Patient Evidence in XLH (APEX) program is a global data unification project that combines 3 ongoing observational, longitudinal (10-year) studies (33). The APEX program aims to describe the burden and lifelong progression of XLH, collect real-world data on treatment effectiveness and safety, and investigate regional differences in treatment outcomes (33). To better understand the impact of burosumab treatment on growth, data from the APEX program were analyzed to assess the impact of treatment over time on growth in burosumab-treated and burosumab-naïve (either treated with Pi/D or entirely untreated) participants with XLH aged 2-17 years.

## Methods

### APEX design

The design of the APEX program has been described in detail (33). In brief, APEX includes 3 observational, noninterventional, retrospective and prospective, multicenter, longitudinal cohort studies of patients with XLH: the International XLH Registry (IXLHR; NCT03193476) collecting data from Europe and Israel; the Study of Longitudinal Observation for Patients With X-linked Hypophosphatemic Rickets/Osteomalacia in Collaboration With Asian Partners (SUNFLOWER; NCT03745521) collecting data in Japan and the Republic of Korea; and the XLH Disease Monitoring Program (XLH DMP; NCT03651505) collecting data from the Americas (this analysis included data from Canada and the United States only, as the data from Latin America were not available for the current analysis). APEX harmonized the data from these 3 sources as previously described (33), performing all analyses on the unified datasets after checking values against range limits and verifying consistent, shared information across the 3 protocols.

### Data collection

Regional study data were collected via purposefully designed electronic case report forms, with data stored according to local regulations to maintain data protection. Retrospective (up to 5 years prior to enrollment) and baseline data were collected at enrollment in 1 of the 3 respective regional studies; prospective (postenrollment) data were collected at routine clinical visits. Prospective data were included for the following years based on time of enrollment: IXLHR 2017-2024; SUNFLOWER 2018-2024, XLH DMP 2018-2024.

### Ethics

The 3 studies were conducted in accordance with local legislation and institutional requirements. All participants (or their legal guardians) provided written informed consent, or assent where required, before inclusion in the individual registries and APEX.

### Study population

**Figure 1:**
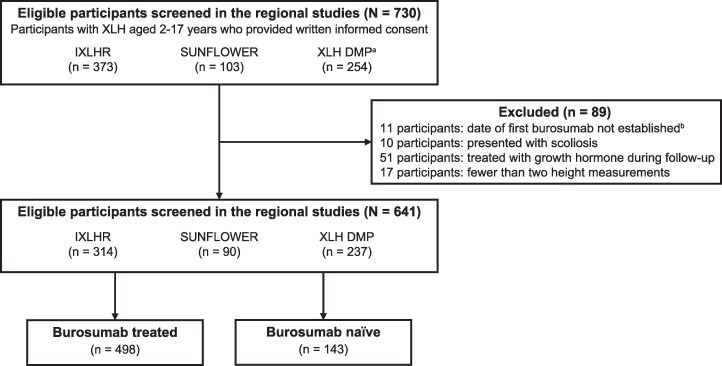
Patient selection flowchart. ^a^Latin American participant data were not available for this analysis. ^b^Some of these patients were removed because they may have experienced burosumab use not recorded in the APEX data as a part of the SUNFLOWER regional study. APEX, Advancing Patient Evidence in XLH; IXLHR, International XLH Registry; SUNFLOWER, Study of Longitudinal Observation for Patients With X-linked Hypophosphatemic Rickets/Osteomalacia in Collaboration With Asian Partners; XLH, X-linked hypophosphatemia; XLH DMP, XLH Disease Monitoring Program.

All eligible participants in the individual studies were diagnosed with XLH, provided written informed consent, and were not participating in a clinical trial at the time (or had received prior approval to do so in the XLH DMP). As enumerated in Fig. 1, participants were excluded from the current analysis if they had <2 height measurements between the ages of 2-17 years during the follow-up period; had a record of scoliosis of any degree of severity; were treated with growth hormone at any point during follow-up; or did not have a date of treatment initiation. Participants who had undergone osteotomy were not excluded from this analysis (as burosumab treatment may affect rates of osteotomy surgeries; therefore, osteotomy may be a mediator of the burosumab effect on growth rather than a confounder).

Eligible participants were categorized as either burosumab treated or burosumab naïve. Participants classified as “burosumab treated” could have been untreated or treated with Pi/D prior to initiating burosumab. Burosumab-naïve participants were either treated with Pi/D or entirely untreated. As pediatric participants aged <1 year are not eligible for burosumab treatment in some regional studies, and the height data included “lag times” of up to 1 year (see Statistical Analysis), participants aged <2 years at enrollment were excluded from this analysis. Furthermore, growth in participants with XLH aged <2 years appears to follow a different overall trend than the generally linear growth seen in children aged 2­-13 years without XLH (12).

### Statistical analysis

Height Z-scores were calculated based on the Box–Cox transformation. The age-specific parameters used to calculate the height Z-scores for non-Asian participants were obtained from the US Centers for Disease Control and Prevention (CDC) (34). Age-specific parameters used to calculate height Z-scores for Asian participants were based on Japanese national survey data (35). When including data from both recumbent length and standing height, 0.8 cm was subtracted from the recumbent length, as per the 2000 CDC Growth Charts for the US (36).

Phosphate and alkaline phosphatase (ALP) values used in this analysis were based on the earliest available value within the follow-up period. Phosphate and ALP Z-scores were based on the sex- and age-specific Box–Cox parameters in Pott et al and Wilson et al, respectively (37, 38).

The primary analytical approach was a mixed regression model on patient height that estimated growth velocity as a change in height over time with a random effect for the patient-level intercept (39). Additionally, an autoregressive-1 correlation matrix was used to allow within-subject correlation of height measurements. The growth velocity per participant year was estimated using a covariate for participant age and updated at every height measurement. The effect of burosumab was estimated using an interaction term between participant age and the treatment indicator as outlined in Huang et al (39). “Subjects” were defined by participant and treatment. If a participant initiated burosumab treatment during the study, a new “subject” was created to allow clear differentiation of non-burosumab and burosumab treatment outcomes.

**Figure 2:**
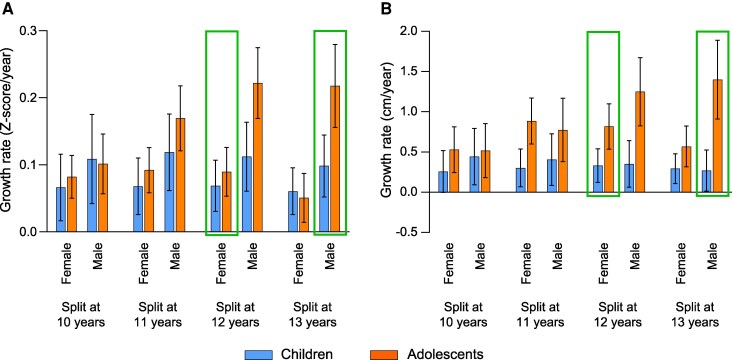
Sensitivity analysis of additional growth velocity among burosumab-treated over burosumab-naïve participants using different puberty age thresholds. Panel A depicts Z-scores, and panel B depicts absolute values. Green boxes identify the splits used in the primary analyses.

Children with XLH generally demonstrate a stable linear growth pattern between the ages of 2 and 13 years (12), but it cannot be assumed that growth continues in a strictly linear fashion after puberty. Therefore, children and adolescents were modeled separately in this analysis. To determine the most appropriate age thresholds for puberty in female and male participants, the mean age at enrollment of the included participants was investigated for each Tanner stage (Table 1). As a result, we used separate puberty age thresholds for females (children aged 2-11 years; adolescents aged 12-17 years) and males (children aged 2-12 years; adolescents aged 13-17 years). The age threshold for males and females corresponded roughly to the age between Tanner stages 2 and 3. As the age at which patients transition from childhood to adolescence varies by patient, sensitivity analyses varying the threshold ages used in these definitions were conducted. Sensitivity analyses using puberty threshold ages ranging from 10 to 13 years showed burosumab treatment was associated with a greater growth irrespective of the threshold used (Fig. 2).

**Table 1:** Tanner stage at enrollment

	Females		Males		Mean age difference, years
Tanner stage	*n* (%)	Mean age, years	*n* (%)	Mean age, years	
1	169 (42)	6.4	141 (59)	7.1	0.7
2	28 (7)	10.8	16 (7)	11.9	1.1
3	15 (4)	13.1	11 (5)	13.3	0.2
4	31 (8)	14.2	8 (3)	15.4	1.2
5*^a^*	48 (12)	15.0	12 (5)	14.8	−0.2
Not reported	111 (28)	8.4	51 (21)	7.9	−0.5

^*a*^Tanner stage 5 includes participants with a reported Tanner stage 6.

A small number of participants were reported as Tanner stage 6 by clinical site investigators. As this Tanner stage was not reported in all the regional studies and is in general not commonly used, all participants with a reported Tanner stage 6 were included in the Tanner stage 5 group.

**Figure 3:**
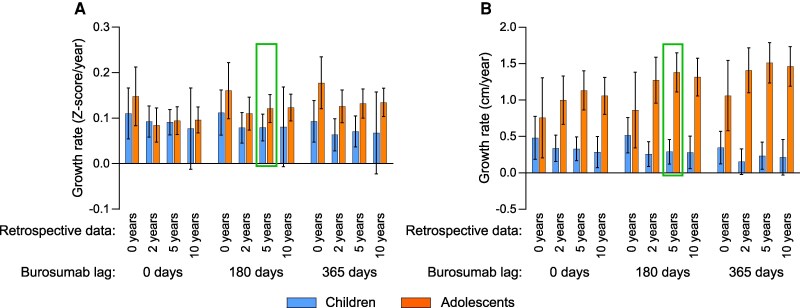
Analysis of participant's growth velocity by varying periods of time for lag/retrospective data decisions. Panel A depicts Z-scores, and panel B depicts absolute values. Error bars depict 95% confidence intervals. Green boxes highlight the data used in the primary analyses.

The effects of burosumab on participant growth velocity were assessed using varying periods of time for the retrospective/pre-enrollment data (0-10 years) and varying lag times from the initiation of burosumab to the analysis of the burosumab effect on growth (0-365 days; Fig. 3). The primary analysis used 5 years of retrospective data, as these data were more complete. A 180-day lag between the initiation of burosumab treatment and the modeled onset of the burosumab effect was used. This approach allowed burosumab dose optimization and prevented any atypical early growth from biasing the long-term growth estimates. The 180-day lag generally produced results between those of the 0-day lag and 365-day lag models and was used for the primary analysis. The estimated association between burosumab and growth remained positive in all analyses, with increased effect of burosumab seen with increased lag, indicating that the conclusions are not unduly sensitive to the amount of retrospective data included or the length of the lag period.

Data were analyzed in a manner parallel to the intent-to-treat analyses used in RCTs—once a participant was considered part of the burosumab treatment group (ie, started burosumab at least 180 days prior), they were not removed from the burosumab treatment group if they stopped or paused burosumab treatment. In the adjusted models, adjustments were made for age, regional study, race, phosphate Z-score, ALP Z-score, and Tanner stage.

Continuous variables were summarized by arithmetic mean with SD and median with interquartile range (IQR) or range. Categorical variables were summarized by frequencies and proportion of participants. Statistical tests were all 2-sided and a *P* value <.05 was considered significant. All analyses were conducted using the SAS Enterprise Guide version 8.3 (SAS Institute Inc., Cary, NC, USA).

## Results

The data cut was June 2024 in IXLHR; December 2023 in SUNFLOWER; and February 2024 in XLH DMP. A total of 1557 participants were enrolled in APEX by the cutoff dates; 730 participants aged 2-17 years had provided informed written consent, with 641 eligible for inclusion in this study (Fig. 1).

Retrospective height records before enrollment span a median (IQR) of 2.8 (0.3-4.4) years for burosumab-treated and 2.6 (0.5-4.1) years for burosumab-naïve participants. We included 2992 retrospective height measurements and 2211 prospective height measurements in this analysis. The median (IQR) height measurement follow-up (combining retrospective plus prospective records) was 4.9 (3.2-7.5) years for burosumab-treated participants and 5.1 (2.9-6.6) years for burosumab-naïve participants. Among burosumab-treated patients, the median treatment duration (ie, difference between first and last height date after burosumab initiation) was 3.0 years.

**Figure 4:**
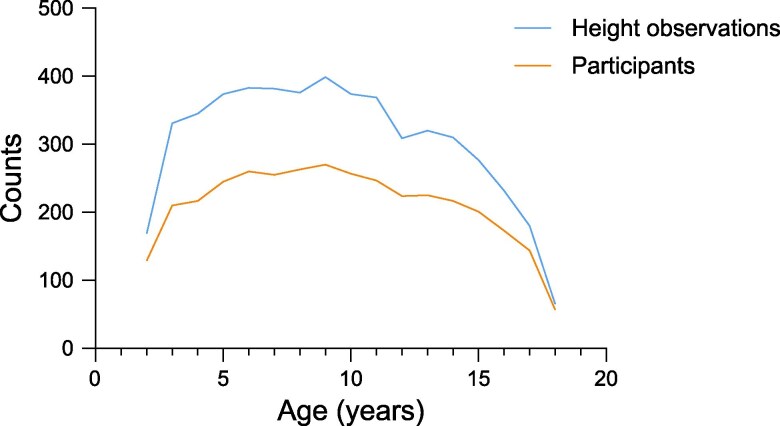
Counts of height observations and participants with at least one height observation within each year of age.

Overall, 5203 qualifying height measurements were recorded. Imputing a height measurement when a participant had a height measurement within 1 year of the sex-specific threshold added 277 height observations for a total of 5480 height measurements. The mean number of height measurements per participant was 8.5 and the median was 7.0 (IQR: 4 to 11). The number of height measurements per participant ranged from 2 to 36. Height measurements were most frequently collected for participants aged 3-12 years (Fig. 4).

Most eligible participants were treated with burosumab (*n* = 498 [78%]) and were from IXLHR (*n* = 234 [47%]) and XLH DMP (*n* = 217 [44%]), with SUNFLOWER contributing 47 (9%) burosumab-treated participants (Table 2). Of the burosumab-treated participants, 372 (75%) were children and 126 (25%) were adolescents at enrollment. The median (IQR) enrollment ages were 7 (4-9) years for burosumab-treated and 5 (3-9) years for burosumab-naïve children (children were defined as females aged 2-11 years and males aged 2-12 years), and 14 (13-16) years for burosumab-treated and 15 (14-16) years for burosumab-naïve adolescents (adolescents were defined as females aged 12-17 years and males aged 13-17 years). Among children, the majority were Tanner stage 1 in both the burosumab-treated group (*n* = 247 [66%]) and burosumab-naïve group (*n* = 49 [55%]) at enrollment. Among burosumab-treated adolescents, more were in Tanner stages 2-4 (*n* = 49 [39%]) than in Tanner stage 5 (*n* = 38 [30%]), while the proportions in burosumab-naïve adolescents were roughly equal (Tanner stages 2-4: *n* = 15 [28%]; Tanner stage 5: *n* = 19 [35%]). Tanner stage was not reported for 114 (23%) burosumab-treated and 48 (34%) burosumab-naïve participants. As our approach was an intent-to-treat analysis, examination of the effects of discontinuation of therapy was outside the scope of the project; however, we estimated that discontinuation events >180 days occurred in fewer than 50 of the 498 treated patients. Of these 50 patients, only 4 had a reason for discontinuation recorded: 2 were “physician decision” and 2 were “lack of effectiveness.”

**Table 2:** Participant enrollment characteristics

	Burosumab-treated participants			Burosumab-naïve participants			Total
	Children	Adolescents	All	Children	Adolescents	All	All
	(*n* = 372)	(*n* = 126)	(*n* = 498)	(*n* = 89)	(*n* = 54)	(*n* = 143)	(*N* = 641)
Registry, *n* (%)							
IXLHR	187 (50)	47 (37)	234 (47)	55 (62)	25 (46)	80 (56)	314 (49)
SUNFLOWER	31 (8)	16 (13)	47 (9)	25 (28)	18 (33)	43 (30)	90 (14)
XLH DMP	154 (41)	63 (50)	217 (44)	9 (10)	11 (20)	20 (14)	237 (37)
Age, years, median (IQR) [*n*]	7 (4-9) [372]	14 (13-16) [126]	8 (5-12) [498]	5 (3-9) [89]	15 (14-16) [54]	10 (4-14) [143]	9 (5-13) [641]
Female, *n* (%)	219 (59)	89 (71)	308 (62)	50 (56)	44 (81)	94 (66)	402 (63)
Phosphate Z-score, median (IQR) [*n*]	−3.7 (−4.7 to −2.5) [351]	−2.4 (−3.2 to −1.5) [123]	−3.2 (−4.5 to −2.2) [474]	−4.8 (−5.6 to −3.7) [82]	−2.9 (−3.6 to −2.4) [51]	−3.9 (−5.1 to −2.9) [133]	−3.3 (−4.7 to −2.4) [607]
<−3, *n* (%)	223 (60)	36 (29)	259 (52)	71 (80)	24 (44)	95 (66)	354 (55)
−3 to −2.1, *n* (%)	72 (19)	43 (34)	115 (23)	8 (9)	21 (39)	29 (20)	144 (22)
≥−2, *n* (%)	56 (15)	44 (35)	100 (20)	3 (3)	6 (11)	9 (6)	109 (17)
Phosphate, mg/dL, median (IQR) [*n*]	3.0 (2.6-3.6) [351]	2.9 (2.4-3.4) [123]	3.0 (2.5-3.5) [474]	2.6 (2.3-3.1) [82]	2.6 (2.3-2.9) [51]	2.6 (2.3-3.0) [133]	2.9 (2.5-3.5) [607]
Not reported, *n* (%)	21 (6)	3 (2)	24 (5)	7 (8)	3 (6)	10 (7)	34 (5)
ALP Z-score, median (IQR) [*n*]	2.2 (1.0-3.2) [371]	1.9 (0.8-2.8) [124]	2.1 (1.0-3.2) [495]	2.7 (1.6-4.0) [84]	1.9 (1.0-3.7) [50]	2.4 (1.5-3.9) [134]	2.1 (1.1-3.3) [629]
≥3, *n* (%)	114 (31)	27 (21)	141 (28)	37 (42)	13 (24)	50 (35)	191 (30)
2-2.9, *n* (%)	80 (22)	32 (25)	112 (22)	20 (22)	11 (20)	31 (22)	143 (22)
<2, *n* (%)	177 (48)	65 (52)	242 (49)	27 (30)	26 (48)	53 (37)	295 (46)
ALP, U/L, median (IQR) [*n*]	408 (312-531) [371]	275 (183-447) [124]	386 (278-505) [495]	476 (371-589) [84]	279 (196-402) [50]	400 (302-539) [134]	388 (279-511) [629]
Not reported, *n* (%)	1 (0)	2 (2)	3 (1)	5 (6)	4 (7)	9 (6)	12 (2)
Race/ethnicity, *n* (%)							
White	223 (60)	77 (61)	300 (60)	34 (38)	19 (35)	53 (37)	353 (55)
Asian	37 (10)	19 (15)	56 (11)	25 (28)	20 (37)	45 (31)	101 (16)
Black	8 (2)	1 (1)	9 (2)	0	1 (2)	1 (1)	10 (2)
Hispanic	29 (8)	9 (7)	38 (8)	6 (7)	4 (7)	10 (7)	48 (7)
Other/unknown race*^a^*	75 (20)	20 (16)	95 (19)	24 (27)	10 (19)	34 (24)	129 (20)
Tanner stage, *n* (%)							
1	247 (66)	10 (8)	257 (52)	49 (55)	4 (7)	53 (37)	310 (48)
2**-**4	38 (10)	49 (39)	87 (17)	7 (8)	15 (28)	22 (15)	109 (17)
5	2 (1)	38 (30)	40 (8)	1 (1)	19 (35)	20 (14)	60 (9)
Not reported	85 (23)	29 (23)	114 (23)	32 (36)	16 (30)	48 (34)	162 (25)
Height							
Z-score, median (IQR) [*n*]	−1.5 (−2.3 to −0.7) [372]	−1.8 (−2.7 to −1.1) [126]	−1.6 (−2.4 to −0.8) [498]	−1.3 (−1.8 to −0.5) [86]	−1.6 (−2.3 to −0.9) [54]	−1.3 (−2.1 to −0.7) [143]	−1.5 (−2.3 to −0.7) [638]
cm, median (IQR) [*n*]	112 (98-127) [372]	148 (143-155) [126]	122 (104-140) [498]	108 (93-129) [89]	150 (146-156) [54]	131 (98-147) [143]	123 (103-143) [638]

Percentages may not add up to 100% due to rounding. Children are female participants aged 2-11 years and male participants aged 2-12 years. Adolescents are female participants aged 12-17 years and male participants aged 13-17 years.

Abbreviations: ALP, alkaline phosphatase; IQR, interquartile range; IXLHR, International XLH Registry; SUNFLOWER, Study of Longitudinal Observation for Patients With X-linked Hypophosphatemic Rickets/Osteomalacia in Collaboration With Asian Partners; XLH, X-linked hypophosphatemia; XLH DMP, XLH Disease Monitoring Program.

^*a*^Race data were not reported in all countries.

**Figure 5:**
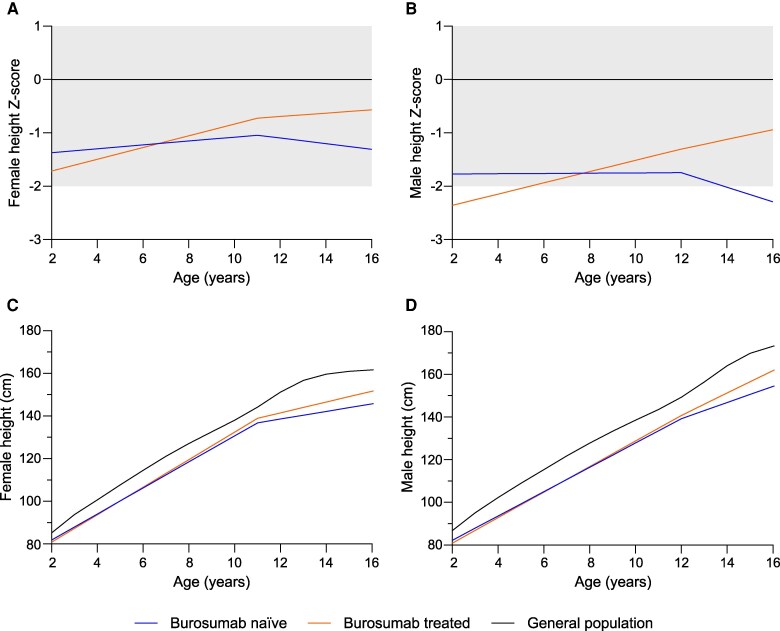
Predicted height adjusted growth trajectories for burosumab-treated and burosumab-naïve participants throughout their development according to Z-score (panels A and B) and cm (panels C and D) for female (panels A and C) and male (panels B and D) participants. Orange lines represent burosumab-treated participants, blue lines represent burosumab-naïve participants, and gray lines represent reference populations. The shaded area of panels A and B represents the normal range of height Z-scores (+/− 2 Z-scores), with zero representing the median height of the reference population. Gray lines in panels C and D represent the median height of the reference population. The reference population for absolute values (panels C and D) consists of weighted averages between the CDC and Japanese national data, in accordance with the proportion of Asian participants among male APEX patients (10.5%) and female APEX patients (18.9%). Note, linear models that assumed constant growth within each time period were used. The straight lines in each graph represent this estimated average growth during the entire period. We do not claim that growth itself is linear throughout the time periods, and acknowledge, for example, that 17-year-olds do not grow at the same pace as 14-year-olds; we are comparing covariate-adjusted model results in order to estimate the overall impact of burosumab treatment on growth within each time period. APEX, Advancing Patient Evidence in XLH; CDC, US Centers for Disease Control and Prevention.

**Figure 6:**
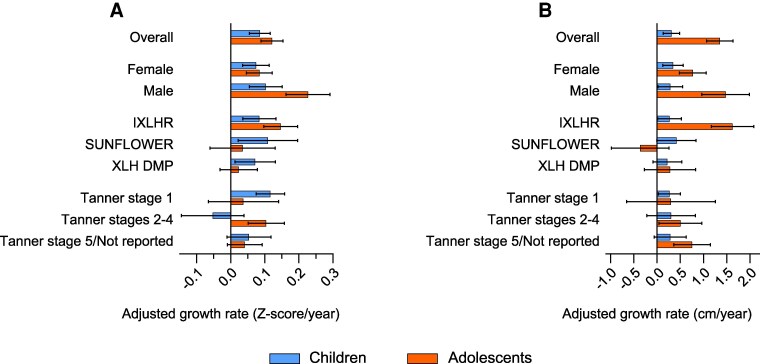
Subgroup analysis of the adjusted additional growth velocity among burosumab-treated over burosumab-naïve participants. Panel A depicts Z-scores, and panel B depicts absolute values. Error bars depict 95% confidence intervals. Adjusted for registry, sex, initial serum phosphate Z-score, initial serum ALP Z-score, race, and Tanner stage. Children are female participants aged 2-11 years and male participants aged 2-12 years. Adolescents are female participants aged 12-17 years and male participants aged 13-17 years. ALP, alkaline phosphatase; IXLHR, International XLH Registry; SUNFLOWER, Study of Longitudinal Observation for Patients With X-linked Hypophosphatemic Rickets/Osteomalacia in Collaboration With Asian Partners; XLH, X-linked hypophosphatemia; XLH DMP, XLH Disease Monitoring Program.

Using regression models based on Z-score/year and cm/year, both child and adolescent participants treated with burosumab, vs those who were burosumab naïve, experienced statistically significantly faster growth (Table 3). Overall, among participants treated with burosumab, improved growth vs burosumab-naïve participants was more pronounced in adolescents than in children. After full adjustment for all described variables, growth in children was greater in burosumab-treated participants than in burosumab-naïve participants by height Z-score/year of 0.085 (95% confidence interval [CI]: 0.055 to 0.116; *P* < .0001) and by cm/year of 0.31 (95% CI: 0.13 to 0.48; *P* = .0007). In adolescents, growth was greater in burosumab-treated participants than in burosumab-naïve participants by height Z-score/year of 0.121 (95% CI: 0.089 to 0.154; *P* < .0001) and by cm/year of 1.35 (95% CI: 1.07 to 1.63; *P* < .0001), in the fully adjusted model. Predicted child height Z-scores in the model increased for both burosumab-treated and burosumab-naïve participants. Note that baseline height Z-scores for burosumab-treated participants were lower compared with burosumab-naïve participants and thus, the model predicts a lower starting height for burosumab-treated participants vs burosumab-naïve participants. Burosumab-treated participants become taller than burosumab-naïve participants at approximately 5 and 7 years of age for females and males, respectively. Predicted adolescent height Z-scores in the model continued to increase in both female and male burosumab-treated participants, whereas they decreased in burosumab-naïve participants (Fig. 5A and 5B). Correspondingly, predicted absolute height increased in both burosumab-treated and burosumab-naïve participants, but the trajectory of increase was greater in those who received burosumab (Fig. 5C and 5D). This difference was more pronounced in adolescent participants. Thus, the modeling predicted adolescent height among burosumab-treated participants was closer to sex-specific general population height than in burosumab-naïve participants (34). In burosumab-treated participants, modeling predicted a similar growth trajectory prior to puberty and an increased growth trajectory after puberty in males vs females, as assessed by both Z-scores/year and cm/year (Figs. 5 and 6). Overall, based on the age group and sex-specific regression estimates, burosumab-treated participants were projected to be taller than burosumab-naïve participants at the end of growth. For example, the model predicts that a hypothetical male patient treated with burosumab throughout ages 2 to 17 may have a height advantage of 10 cm (172 cm compared with 162 cm) compared to a hypothetical male patient who was burosumab naïve. For hypothetical female patients, the estimated additional effect of burosumab treatment throughout ages 2 to 17 may be 8 cm (157 cm compared with 149 cm).

**Table 3:** Regression model determined additional growth of burosumab-treated participants vs burosumab-naïve participants

		Unadjusted	Adjusted for registry and sex	All adjustments
Height: Z-score/year				
Children	Estimate (95% CI)	0.082 (0.050-0.113)	0.084 (0.053-0.115)	0.085 (0.055-0.116)
	*P* value	<.0001	<.0001	<.0001
Adolescents	Estimate (95% CI)	0.133 (0.101-0.165)	0.128 (0.096-0.160)	0.121 (0.089-0.154)
	*P* value	<.0001	<.0001	<.0001
Height: cm/year				
Children	Estimate (95% CI)	0.31 (0.13-0.49)	0.32 (0.14-0.49)	0.31 (0.13-0.48)
	*P* value	.0008	.0003	.0007
Adolescents	Estimate (95% CI)	1.43 (1.16-1.70)	1.39 (1.11-1.67)	1.35 (1.07-1.63)
	*P* value	<.0001	<.0001	<.0001

Registry adjustment adjusts for IXLHR, SUNFLOWER, or XLH DMP. All adjustments adjust for registry, sex, initial serum phosphate Z-score, initial serum ALP Z-score, race, and Tanner stage. Children were female participants aged 2-11 years and male participants aged 2-12 years. Adolescents were female participants aged 12-17 years and male participants aged 13-17 years.

Abbreviations: ALP, alkaline phosphatase; CI, confidence interval; IXLHR, International XLH Registry; SUNFLOWER, Study of Longitudinal Observation for Patients With X-linked Hypophosphatemic Rickets/Osteomalacia in Collaboration With Asian Partners; XLH, X-linked hypophosphatemia; XLH DMP, XLH Disease Monitoring Program.

Subgroup analyses showed positive incremental annual growth across most of the subgroups, indicating that burosumab-treated participants experienced higher growth velocities than burosumab-naïve participants (Fig. 6). Consistent with the main analysis, incremental annual growth was greater in adolescent males (0.2 Z-score/year and 1.4 cm/year) than adolescent females (0.1 Z-score/year and 0.8 cm/year). Negative associations with annual cm-based growth were observed for adolescent participants from the SUNFLOWER study and Z-score-based growth among child participants in Tanner stages 2 to 4. However, these subgroups had small sample sizes, resulting in data that were highly variable with wide confidence intervals.

A sensitivity analysis excluding the 57 patients who underwent an osteotomy during follow-up (35 of whom were in the burosumab-treated cohort) reached substantially similar results as the primary analysis. The overall Z-score effects were 0.085 for younger participants (*P* < .0001) and 0.12 for adolescents (*P* < .0001; Table 3). When patients who underwent osteotomies were excluded, these results were 0.091 for younger participants (*P* < .0001) and 0.13 for adolescents (*P* < .0001; data not shown).

## Discussion

This analysis represents the first longitudinal report of unified pediatric global growth data from the APEX program. Participants treated with burosumab experienced significantly greater growth velocity than burosumab-naïve participants, as well as an increase in growth compared with reference populations, demonstrating the real-world effectiveness of burosumab treatment in children and adolescents over a follow-up of approximately 5 years. Projections of final height were greater in burosumab-treated than burosumab-naïve participants, albeit still lower than the median adult height in the general population.

We report that burosumab-treated patients grew faster than burosumab-naïve patients by 0.082 Z-score/year among children and 0.121 Z-score/year among adolescents. These results are generally consistent with RCTs and real-world studies, which showed that XLH patients treated with burosumab experienced greater improvements in height Z-score as compared to those treated with Pi/D (21, 23-30, 40). However, changes in growth velocity or Z-scores have not been apparent in all studies of burosumab (31, 32). Rather, in some reports, burosumab treatment appeared to prevent reductions in growth velocity that typically occur with Pi/D (3).

This study provides a meaningful statistical analysis of the impact of burosumab treatment on growth in adolescents, in whom no meaningful prior analysis has been available, due to the exclusion of adolescents from burosumab RCTs; furthermore, the number of adolescent participants in real-world studies has been limited. In the APEX study, regression modeling indicated that burosumab treatment could sustain superior growth compared with burosumab-naïve participants during puberty, mitigating an XLH-related attenuation of the pubertal growth spurt. We hypothesize that Pi/D is unable to sustain the adolescent growth spurt because it does not adequately resolve the hypophosphatemia-associated mineralization defect during a time of increased mineral demand (23, 31). Additionally, low adolescent growth rates may be due to reduced adherence of Pi/D in that age group (41), although it was not possible to assess medication adherence in this study, due to its real-world nature. One study of pediatric patients with XLH treated with Pi/D, where therapy was controlled and monitored, reported a progressive decrease in growth velocity throughout childhood, with a marked decrease in Z-scores by the time of the expected pubertal growth spurt (9). Thus, even with good adherence, Pi/D appears to be unable to maintain normal adolescent growth.

Greater growth among burosumab-treated participants compared with burosumab-naïve participants occurred across most of the subgroups evaluated, including subcohorts of male and female participants, the IXLHR and XLH DMP regional studies, and a prepubertal (Tanner stage 1) subcohort. The negative associations observed for participants in cm-based analyses of adolescent SUNFLOWER patients may be due to the absence of retrospective burosumab growth data in this registry, resulting in a shorter study period. It is also possible that the effects may be simple random variation.

Burosumab may largely improve growth by healing rickets and improving lower limb deformities, resulting in straighter legs. Burosumab was superior to Pi/D at treating rickets, with improvement on the Radiographic Global Impression of Change scale and greater reductions in Rickets Severity Scale scores and serum ALP (23). Switching from Pi/D to burosumab significantly corrects lower limb malalignment in children (42, 43). Interestingly, more severe malalignment was associated with lower height Z-scores, while lower height Z-scores were also associated with greater correction in lower limb alignment (42), supporting this hypothesis. Based on X-ray imaging, treatment with burosumab improves measures of rickets prior to observed correction of lower limb alignment in children (21, 23, 24, 42, 43). Examination of potential factors on growth independent of the consequences of healing rickets would be of interest to consider in future studies, albeit these treatment effects are highly confounded.

Different growth characteristics have been described in studies analyzing the effects of burosumab. One RCT found that 1- to 4-year-old children with XLH had an initial increase in growth velocity after 24 weeks of burosumab treatment that subsequently plateaued, whereas children aged 5-12 years demonstrated a more consistent increase in growth velocity over the 64-week treatment period (24). In the follow-up study, the crossover group, initially treated with Pi/D for 64 weeks, did not show an increase in growth velocity when transitioned to burosumab during weeks 64 to 84 (44). In an RCT extension study, sustained growth velocity was seen in children aged 5-12 years up to 160 weeks, with a trend for greater growth velocities in the initial 40 weeks (40). A real-world, retrospective, single-center study described a relatively consistent increase in growth velocity in 12 pediatric patients aged 2-18 years over a 2-year period of burosumab treatment, with the largest increase in growth velocity in the first 6 months (29). Additionally, 2 clinical trials did not demonstrate any increase in growth velocity from baseline in burosumab-treated patients (31, 32). Differences in these findings may be due to individual patient differences and small numbers of participants, but they may also represent specific age-related differences in growth of children with XLH (8, 9). The current analysis does not report on the effect of burosumab treatment at specific time points after burosumab initiation. Rather, it uses regression modeling to generate an estimate of the average expected growth, assuming the growth velocities observed would have continued for the full duration of burosumab treatment. Nevertheless, the sensitivity analyses using lag times after onset of treatment indicate that the burosumab impact on growth velocity is similar when 0-, 180-, and 365-day lag times are employed, suggesting that the initial increase in growth velocity following onset of burosumab treatment, as reported in some studies, was not replicated here.

Previous studies of Pi/D and burosumab support the use of early treatment (ie, during infancy) to maximize height gain (10-13, 30). Earlier initiation and longer duration of burosumab treatment may support starting the pubertal growth spurt at a greater height and further enhance final adult height. Of all participants included in this analysis, 71 patients presumably reached adult height (at least one measured height at or after age 14 for females and age 16 for males) and had received burosumab treatment at least 180 days prior to that height measurement (median: 2.1 years prior). However, it was not possible to map the true effect of treatment with burosumab on growth or final height or with respect to early initiation as no participants were treated from the earliest possible age of initiation into adulthood.

The determination of the age thresholds of puberty for male and female participants at ages 13 and 12, respectively, were based on the averages of a wide range of participants. Therefore, these puberty thresholds do not capture individual variability regarding the age at which their puberty begins; however, sensitivity analyses varying these thresholds from 10 to 13 years did not substantially change the conclusions regarding the positive effects of burosumab. However, despite the relatively large data set, there were too few participants to model the adolescent growth spurt and the subsequent slowing of growth that occurs as participants near their final height. Thus, a linear model of adolescent growth was utilized to describe the average predicted growth of participants with XLH over their adolescence.

Because APEX and the 3 regional studies report data collected during routine clinical visits, some questions about the effects of burosumab on growth currently remain unanswered. First, patients with XLH who exhibit more severe symptomology may be more likely to be selected for treatment with burosumab (45). This may have had an impact on growth among burosumab-treated participants in this study. Second, participants who underwent osteotomy were included in this study. It is not known whether the need for osteotomy is influenced by burosumab, how much the procedure impacts height, or how height is affected by the age at which osteotomy occurs. A sensitivity analysis excluding patients who underwent an osteotomy during follow-up showed similar results to the primary analysis, suggesting osteotomy did not affect the overall results. Finally, participants with recorded scoliosis were excluded from this analysis; however, collection of this information was not systematic across the studies. Therefore, some participants with scoliosis may have been included inadvertently.

Limitations of the study include the varying numbers of height observations per participant (range, 2 to 36), the unknown effect of previous treatment with Pi/D on growth after burosumab initiation, and several factors that may influence the estimation of the impact of burosumab on growth. Indeed, several elements of the study design may have affected the estimate of change in height with burosumab treatment. First, the degree of treatment adherence among participants was unknown. Second, an intent-to-treat-style analysis was applied, in which participants were retained in the analysis even if they stopped or had intermittent burosumab treatment—analyses limited to time periods where patients were adherent to Pi/D or burosumab were beyond the scope of this study. Third, this analysis compared burosumab-treated with all burosumab-naïve participants, which was a combined group of Pi/D-treated and untreated participants. Due to the low number of participants who were untreated, it was not feasible to analyze the Pi/D-treated and untreated participants separately. The baseline APEX analysis, a separate analysis with a nearly identical pediatric population, reported that 7% of 1- to 12-year-olds and 10% of 13- to 17-year-olds were untreated at enrollment in APEX (46). Thus, we expected similar proportions of the participants in this study to be untreated. Fourth, participants younger than 2 years were not included in the analysis, preventing modeling of the effect of early treatment initiation on growth. Finally, as the duration of follow-up was limited in this analysis, it is not known whether improvements in growth velocity will be maintained with long-term treatment. Limited follow-up periods are also a weakness of clinical trials investigating the impact of burosumab on growth velocity (31, 32, 40, 44).

## Conclusion

This study of growth in pediatric participants in the APEX program combines data from 3 regional observational studies, establishing the largest cohort of patients with XLH currently available. Our unique analysis employs a harmonized data set to assess the impact of burosumab treatment on growth. The results indicate that burosumab treatment has a robust and positive association with improved growth in both male and female children and adolescents under routine clinical care for XLH across multiple regions of the world. Modeling predicts burosumab treatment of children and adolescents with XLH, compared with no burosumab treatment, can support enhancement of growth that will likely result in greater adult height. Future analyses from APEX should provide long-term assessments of final height, may provide sufficient data to test the modeling predictions included in this analysis, and may be able to assess other factors that can influence growth/height such as severity of symptoms, dose and duration of treatment, prior treatment(s), age at treatment initiation, and age at puberty.

## Data Availability

The anonymized data used in the current analysis are available from the corresponding author upon reasonable request and after review by a steering committee led by the study sponsor.

## Clinical trial information

The International XLH Registry (IXLHR; NCT03193476); the Study of Longitudinal Observation for Patients With X-linked Hypophosphatemic Rickets/Osteomalacia in Collaboration With Asian Partners (SUNFLOWER; NCT03745521); and the XLH Disease Monitoring Program (XLH DMP; NCT03651505).

## Abbreviations

ALP: alkaline phosphatase
APEX: Advancing Patient Evidence in XLH
CDC: US Centers for Disease Control and Prevention
CI: confidence interval
FGF23: fibroblast growth factor 23
IQR: interquartile range
IXLHR: International XLH Registry
*PHEX*: phosphate-regulating endopeptidase homolog, X-linked
Pi/D: phosphate salts and active vitamin D analogs
RCT: randomized controlled trial
SUNFLOWER: Study of Longitudinal Observation for Patients With X-linked Hypophosphatemic Rickets/Osteomalacia in Collaboration With Asian Partners
XLH: X-linked hypophosphatemia
XLH DMP: XLH Disease Monitoring Program
